# Selenium in Surgery

**DOI:** 10.7759/cureus.72168

**Published:** 2024-10-22

**Authors:** Haytham Alarfaj

**Affiliations:** 1 College of Medicine, King Faisal University, Al-Ahsa, SAU

**Keywords:** cancer, colon, post-surgery, selenium, thyroid

## Abstract

Selenium, a micronutrient essential for many enzymatic functions, is crucial for maintaining human health. Its presence in the human diet is of paramount importance for metabolism and support of the immune system. Many diseases of surgical importance are related to the level of selenoproteins and their influence on different organs.

The aim of this concise narrative review is to highlight the role of selenium as a trace element in various surgical morbidities, a concept that is often neglected or not well perceived by most surgeons.

## Introduction and background

Selenium is a trace element that plays an important role in living cells. The name is derived from the Greek word Selene, which stands for moon [1]. The initial known discovery of Selenium was in 1817 by a Swedish scientist who was researching the causes of illness among workers dealing with sulfuric acid [2].

It was regarded as a toxic element until 1957, when it was recognized to have a beneficial function in preventing lesions in blood vessels, liver, and muscles in rats and chickens [3-4].

Yet, it was not until the early 1970s that Selenium was considered to play a crucial role in the biochemical functions of living cells [5-7]. A link between serum selenium levels and disease occurrence has been thoroughly investigated [8].

The chemistry of selenium has been explored as an amino acid that exists in body proteins. This amino acid is called selenocysteine. Furthermore, it was also confirmed that this amino acid is detected in the redux enzyme glutathione peroxidase in the form of selenoprotein that plays an important role in various body processes. Proteins have at least one selenocysteine residue in their structures [9]. Although selenium is categorized as a trace element, it plays a paramount role in living cell integrity and wellbeing [8].

Selenium has proved to play an active role in several aspects of human biology. These include the immune, endocrine, reproductive, nervous, muscular, and cardiovascular systems [10]. Its role is interpreted by DNA methyltransferase inhibition [11].

An ideal balanced diet should contain all the necessary nutritional components. There must be a balance between animal and plant sources [12]. Selenium, as well as other trace elements, is present in dietary supplements such as meat, fish, and milk, as well as some plants [13-15]. Nuts, seeds, and legumes are rich sources of selenium [16].

Selenium is an essential nutrient for sustainable human health. It acts as an effective anti-carcinogenic agent due to the incorporation of the amino acid selenocysteine that forms selenoproteins, influencing 25 human genes that actively participate in oxidative stress-cell protection, mainly due to the inflammatory response control by redux [17-18].

Nevertheless, selenium deficiency may induce several human morbidities or even mortalities. It may be a direct cause of myocardial infarction. In addition, its deficiency may negatively influence normal fetal development as well as male infertility [19-22].

Selenium plays an important role in surgical morbidities and cure [23]. Thyroid diseases, colorectal cancer, and post-operative surgical recovery are highly influenced by the serum selenium levels [24-30]. Controversy exists regarding the relationship between serum selenium levels and colonic cancer. It is proposed that the antioxidant effect of selenium may prevent the occurrence of colorectal adenoma lesions and its subsequent colonic cancer. Moreover, others have proposed that selenium may induce a protective effect against colonic cancer recurrence [26, 31-33].

## Review

Selenium and the thyroid gland

The thyroid gland expresses a significantly high concentration of selenium compared to other human organs [34].

Thyroid hormone formation is related to many enzymes such as glutathione peroxidase (GPx) and glutathione deiodinase (GPDs), as well as thioredoxin reductase (TrxR). Selenium constitutes an essential particle in the formation of these enzymes. Therefore, selenium deficiency may lead to oxidative damage due to decreased GPx activity, which is highly accused of impairing thyroid activity. Additionally, low selenium causes an autoimmune process in the thyroid gland. Hence, decreased selenium levels would contribute to the pathogenesis of autoimmune thyroiditis (Graves' disease). Selenium may play a role in the regulation of the cell cycle. Its low concentration has a high impact on the development of thyroid cancer [24, 35-37].

Role of Selenium in Thyroid Autoimmunity

GPx, GPDs, and TrxR are characterized by their oxidoreductase functions [38]. These three elements are related to iodine deficiency and high thyroid-stimulating hormone (TSH) [39]. TrxR and nicotinamide adenine dinucleotide phosphate (NADPH) are the pivotal constitutional elements of the thioredoxin system [40]. Many laboratory, clinical, and epidemiological data have suggested a strong relationship between selenium, iodine, and thyroid hormone metabolism [41].

It was proposed that selenium supplementation may have an inhibitory effect on autoimmune thyroiditis [42]. It was concluded that selenium might protect against autoimmune thyroiditis, especially among pregnant women [43-44]. Nevertheless, selenoproteins are essential to decrease the inflammatory status of the thyroid gland and reduce thyroid autoimmunity [37].

Selenium and Thyroid Cancer

The last decades have witnessed a rapid global increase in thyroid cancer prevalence [45-49].

The relationship between thyroid cancer and selenium levels still entails many debates. It was stated that there is no evidence of the existence of thyroid cancer in relation to selenium intake [34]. Contrarily, others proposed that lower serum selenium levels were noticed among patients with thyroid carcinoma, especially the papillary type. Some authors have discussed its lower level among papillary cancer patients with a higher prevalence among females compared to their male peers. They also suggested that serum selenium has a protective effect among those patients [24]. However, most of the current literature has shown an association between selenium levels and the risk of thyroid cancer [50]. Yet, many studies have shown different actions of selenoproteins regarding the growth of tumor cells as some assumed its inhibitory and fighting actions against tumor cells while others suppose its linkage to the carcinogenesis mechanism. Overall, this notion is not fully clarified as many studies are still needed [51-53].

Selenium and the colon

Selenium influences the human colon in a biphasic way. It may play a role in the prevention and treatment of many benign or even malignant morbidities [54-55].

Inflammatory Bowel Disease (IBD) and Selenium

IBD still pose an enigma not only for management but also for their pathogenesis [56]. They are mainly of two prototypical types: Crohn's disease (CD) and ulcerative colitis (UC). Their prevalence is emerging, especially in the United States and Europe. IBD constitutes a critical health problem regarding complications, the price and availability of medications, quality of life, and patients' social stigma [57]. Recent decades have shown an increasing prevalence of IBD among Arabs and Middle East inhabitants. This may be attributed to changing dietary habits with more consumption of a low-fibrous diet [58].

Selenium is among the food supplements that may influence IBDs as it has an essential role in enhancing normal immune functions [59]. Although the mechanism of IBDs' progression to colonic cancer and the role of selenium in this pathway is not fully understood, many studies have declared an inverse relationship between IBDs and selenium levels [60].

Selenium supplementation is reported to have a protective mechanism against tissue damage induced in UC. This occurs by inducing changes in the expression of certain genes involved in mitochondrial regulation within the dead cells [61]. Nevertheless, selenium may play a beneficial role in modulating inflammation of the gut hand-in-hand with gut microbiota [59]. This mechanism takes place through a cascade of reactions that end up changing macrophage polarization, which induces a significant decrease in gut inflammation [62]. Many studies have suggested a low serum selenium concentration among IBD patients compared to their healthy peers [63-67]. Colectomy, the surgery for treating UC patients, may consequently reduce the serum selenium concentration more than the disease itself. Therefore, post-operative supplementation with selenium-rich food would be necessary [68-69]. Overall, serum selenium concentration is usually lower among CD patients compared to those with UC.

Colon Cancer and Selenium

Some studies have highlighted the role of selenium and its supplementation in different organs' malignancies [70-72].

Selenium is assumed to decrease the size of colonic adenomas, the main precursors for colonic cancer occurrence [73]. Selenoprotein F (SELENOF), one of the currently known selenoproteins, occupies the endoplasmic reticulum organelle and is expressed in many tissues [74]. Many authors have speculated a positive association between altered SELENOF levels and certain neurodegenerative and cancer diseases [75-78]. A trial for cancer prevention involving vitamin E and selenium (SELECT) failed to elucidate a positive role for selenium in prostatic cancer prevention [79]. On the other hand, some studies suggested a protective effect of SELENOF against intestinal malignancies, especially the colon. This is attributed to its effect on the intestinal barrier integrity of the mucosal wall as well as the mucin-producing goblet cells, which may determine the nature and type of the developing tumor [60, 80-83]. Moreover, an animal study proved that low molecular weight selenium compounds may play an effective role in colonic mucosal integrity [84]. It is proposed that selenium may protect the colon against cancer due to its chemopreventive effect through selenoproteins such as glutathione peroxidase (GPx), which may prevent DNA oxidative mutagenic stress [85-88]. Others have suggested effective anticancerous properties of selenium due to their pro-oxidants effects that alter tumor cellular redux homeostasis. It may also prevent metastasis formations efficiently. Hence, many trials are currently investigating its potential chemotherapeutic effect [89-92].

Role of selenium in post-operative and critically ill patients

Selenium has proven to play an important role in post-operative recovery [1, 93]. This may be due to its presence as an essential component in many enzymes' activity [94-101]. It has a crucial role in the process of wound healing as it maintains and regulates many immunological and metabolic reactions in the body [102-103]. Its role in wound healing is mainly during the inflammatory phase by influencing the chemokines and cytokines that play a crucial role during healing [104-108]. Overall, selenoprotein derivatives are crucial during the oxidative stress and inflammation in the cascade of wound healing [109-111]. Yet, excessive selenium levels may have a toxic, unwanted effect on post-operative patients (Selenosis) [112]. On the other hand, many authors have proposed selenium deficiency among surgical patients who were admitted to the intensive care unit post-operatively due to complications [113-118].

## Conclusions

This concise narrative review may have shed light on the importance of selenium in many pathological disorders of general surgeons' interest, as well as the post-operative course for critically ill patients. However, many studies are still needed to investigate this unfulfilled notion of selenium as an important trace element in surgical practice.
